# Genome sequencing of *Mycobacterium pinnipedii* strains: genetic characterization and evidence of superinfection in a South American sea lion (*Otaria flavescens*)

**DOI:** 10.1186/s12864-019-6407-5

**Published:** 2019-12-30

**Authors:** Taiana T. Silva-Pereira, Cássia Y. Ikuta, Cristina K. Zimpel, Naila C. S. Camargo, Antônio F. de Souza Filho, José S. Ferreira Neto, Marcos B. Heinemann, Ana M. S. Guimarães

**Affiliations:** 1 Laboratory of Applied Research in Mycobacteria, Department of Microbiology, Institute of Biomedical Sciences, University of São Paulo, São Paulo, Brazil; 2 Department of Preventive Veterinary Medicine and Animal Health, School of Veterinary Medicine and Animal Sciences, University of São Paulo, São Paulo, Brazil

**Keywords:** *Mycobacterium pinnipedii*, Genome, Superinfection, Comparative genomics, *Mycobacterium tuberculosis* complex

## Abstract

**Background:**

*Mycobacterium pinnipedii*, a member of the *Mycobacterium tuberculosis* Complex (MTBC), is capable of infecting several host species, including humans. Recently, ancient DNA from this organism was recovered from pre-Columbian mummies of Peru, sparking debate over the origin and frequency of tuberculosis in the Americas prior to European colonization.

**Results:**

We present the first comparative genomic study of this bacterial species, starting from the genome sequencing of two *M. pinnipedii* isolates (MP1 and MP2) obtained from different organs of a stranded South American sea lion. Our results indicate that MP1 and MP2 differ by 113 SNPs (single nucleotide polymorphisms) and 46 indels, constituting the first report of a mixed-strain infection in a sea lion. SNP annotation analyses indicate that genes of the VapBC family, a toxin-antitoxin system, and genes related to cell wall remodeling are under evolutionary pressure for protein sequence change in these strains. OrthoMCL analysis with seven modern isolates of *M. pinnipedii* shows that these strains have highly similar proteomes. Gene variations were only marginally associated with hypothetical proteins and PE/PPE (proline-glutamate and proline-proline-glutamate, respectively) gene families. We also detected large deletions in ancient and modern *M. pinnipedii* strains, including a few occurring only in modern strains, indicating a process of genome reduction occurring over the past one thousand years. Our phylogenomic analyses suggest the existence of two modern clusters of *M. pinnipedii* associated with geographic location, and possibly host species, and one basal node associated with the ancient *M. pinnipedii* strains. Previously described MiD3 and MiD4 deletions may have occurred independently, twice, over the evolutionary course of the MTBC.

**Conclusion:**

The presence of superinfection (i.e. mixed-strain infection) in this sea lion suggests that *M. pinnipedii* is highly endemic in this population. *Mycobacterium pinnipedii* proteomes of the studied isolates showed a high degree of conservation, despite being under genomic decay when compared to *M. tuberculosis*. This finding indicates that further genomes need to be sequenced and analyzed to increase the chances of finding variably present genes among strains or that *M. pinnipedii* genome remodeling occurred prior to bacterial speciation.

## Background

Tuberculosis is a contagious and often severe infectious disease caused by members of the *Mycobacterium tuberculosis* complex (MTBC). MTBC is a clonal bacterial group composed of 11 species that can be divided into two major groups: those highly adapted to human beings, named *Mycobacterium tuberculosis* and *Mycobacterium africanum* L5 and L6, and those adapted to animal hosts, named *Mycobacterium bovis, Mycobacterium caprae, Mycobacterium microti, Mycobacterium pinnipedii, Mycobacterium origys, Mycobacterium mungi, Mycobacterium suricattae*, “dassie bacillus”, and “chimpanzee bacillus” [1–3]. These species share great genomic similarity, with more than 99.95% nucleotide identity over alignable regions, and no evidence of horizontal gene transfer or major recombination events [4]. Single nucleotide polymorphisms (SNPs) and deletions of genomic regions ranging from 2 to 12.7 Kb, denominated “regions of difference (RDs)”, allow for species differentiation. Despite this high genetic similarity, members of MTBC vary in their host tropism and ability to cause disease, which are likely consequences of these discrete genetic differences in addition to the presence of duplications and mobile genetic elements [3].

Recently, special attention has been given to the MTBC species known to infect pinnipeds, *M. pinnipedii*, when its DNA was detected in three 1000-year-old, pre-Columbian human skeletons from South America (Peru). This study was the first genetic evidence of tuberculous mycobacteria infecting humans prior to the Europeans’ first contact with the New World [5]. Moreover, it was also the first report of the genome sequencing of modern *M. pinnipedii* strains, yet to be fully compared and analyzed. In modern days, tuberculosis in pinnipeds was first reported in 1913 [6] with *M. pinnipedii* being described as “seal bacillus” in the early 1990s [7, 8] and proposed as a new member of the MTBC in 2003 [2]. Strains of *M. pinnipedii* have been isolated from pinnipeds worldwide, especially in captivity, but also from free-living animals of the southern hemisphere [9, 10]. Surprisingly, since its first description, *M. pinnipedii* has been detected in a variety of host species, including six different pinniped species [11–13], bactrian camels, snow leopards, amur leopards, cattle, llamas, lowland gorillas, Malayan tapirs, Hector’s dolphins, and possibly humans, which supports a generalist behavior for host tropism [9, 10, 14–18]. Pinnipeds infected with *M. pinnipedii* in zoos and marine parks are the main source of infection for other animals and humans. Furthermore, reports of infected cattle were associated with a contaminated water canal connecting directly to the ocean or beach grazing areas where pinnipeds were found [18]. The actual impact, however, of tuberculosis in pinniped species remains largely unknown, mainly because its prevalence in free-ranging seals and sea lions is completely unexplored.

The carcass of a South American sea lion (*Otaria flavescens*) has recently been recovered from the southern coast of the state of Rio Grande do Sul, Brazil. As reproductive colonies of these animals are absent in Brazil, specimens found on the coast are probably from Uruguay. At the animal necropsy, lesions compatible with tuberculosis in different organs were found, from which *M. pinnipedii* isolates were obtained, constituting the first report of this pathogen in the Brazilian coast [19]. Unfortunately, genome sequencing of the isolates was not undertaken at the time, precluding opportunities to better explore the genetic makeup of these bacteria, including the possibility of intra-host bacterial clonal variants (i.e. microevolution) or mixed-strain infection (i.e. superinfection). Until now, *M. pinnipedii* genome sequences have never been fully characterized by comparative genomics. Genomic reads of modern *M. pinnipedii* strains described by Bos et al. [5] were solely used to compare to the ancient *M. pinnipedii*-like genomes. During the development of this study, another strain of *M. pinnipedii* was sequenced [20], but only applied to evaluate the phylogenomic position of this species within the MTBC. Therefore, the objectives of the present study were to sequence and compare two *M. pinnipedii* isolates obtained from different organs of this sea lion carcass [19], and to perform a comparative genomic analysis with other *M. pinnipedii* strains available from public databases.

## Results and discussion

*First description of M. pinnipedii mixed-strain infection in a pinniped.*

**Fig. 1:**
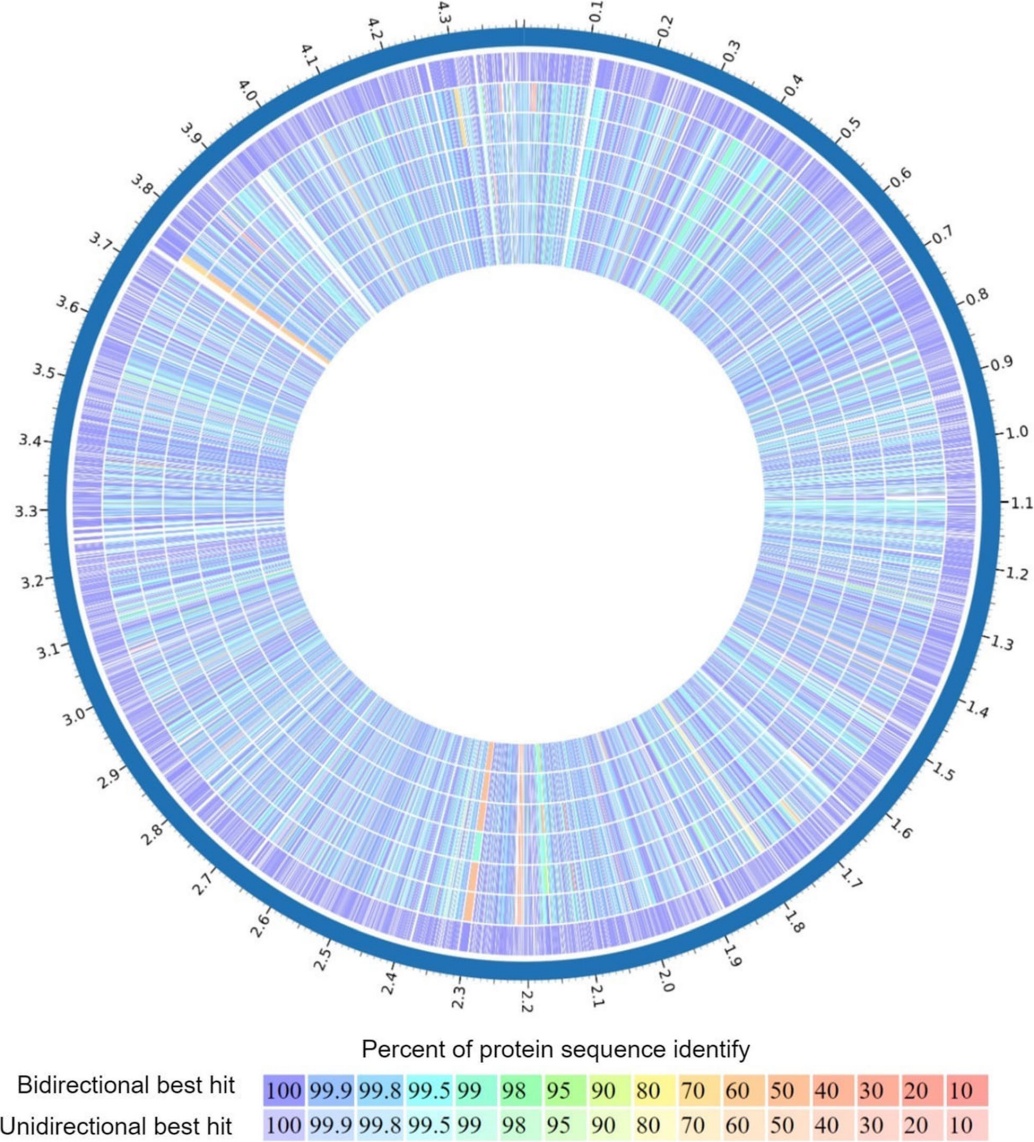
Circular map of proteome of *Mycobacterium pinnipedii* genomes compared to the reference genome of *Mycobacterium tuberculosis* H37Rv. From the outer ring to the inner ring: Genome position, *M. tuberculosis* H37Rv, *M. pinnipedii* MP1, *M. pinnipedii* MP2, *M. pinnipedii* G01222, *M. pinnipedii* G01491, *M. pinnipedii* G01492, *M. pinnipedii* G01498, *M. pinnipedii* ATCC BAA-688. Color scale represents protein identity. Contigs were ordered using MAUVE and map was generated in Patric 3.5.18 web resources

The genome sequencing and assembly of the two *M. pinnipedii* isolates (named MP1 and MP2) resulted in 9,766,154 reads (228x coverage), and 9,433,048 reads (220x coverage), assembled into 102 and 106 contigs (~ 4.28 Mb/genome), respectively. Genome annotation with Prokaryotic Genome Annotation Pipeline (PGAP) indicates a GC content of 65.4% and 45 tRNA for both strains, and 4269 and 4276 genes, 3993 and 3996 coding DNA sequences (CDSs), and 152 and 158 pseudogenes for *M. pinnipedii* MP1 and MP2, respectively. Genomes of both strains showed similar characteristics to other genomes of the same species available in the database (Fig. 1).

Mapping of the *M. pinnipedii* MP1 and MP2 reads with the genome of the *M. tuberculosis* strain H37Rv resulted in a reference coverage of 99.08 and 99.03%, and 2312 and 2350 variants, respectively. Upon exclusion of false-positive prone areas, the isolates shared 1912 SNPs (*M. pinnipedii* MP1) and 1998 SNPs (*M. pinnipedii* MP2) with *M. tuberculosis* H37Rv. In addition, 181 (*M. pinnipedii* MP1) and 208 (*M. pinnipedii* MP2) indels (single-nucleotides or short indels ranging from 2 to 36 bp) were detected. When comparing *M. pinnipedii* MP1 and *M. pinnipedii* MP2, the distance between these strains, after excluding false-positive prone areas, was found to be 113 SNPs and 46 indels (single-nucleotides or short indels ranging from 2 to 36 bp). A total of 122 (76.73%) of these 159 SNPs and indels were located in CDSs with known functions, based on the COG (Cluster of Orthologous Groups) functional annotation (Additional file 1: Table S1). The main functional annotations were those related to energy production and conversion (11.95%, 19/159), lipid transport and metabolism (9.43%, 15/159) and cell wall/membrane/envelope biogenesis (7.55%, 12/159), which are known to play crucial roles in the pathogenicity of MTBC species. Interestingly, the majority of detected SNPs are non-synonymous (86.72%, 98/113), which means that related CDSs are under selective pressure for protein sequence change, having the potential to promote functional diversity.

CDSs of known function with non-synonymous SNPs identified between *M. pinnipedii* MP1 and *M. pinnipedii* MP2 were evaluated using GO enrichment analysis and STRING protein networks. The statistically significant biological processes identified were: trehalose metabolic process (*P* value = 3.07E-04), fatty acid biosynthetic process (P value = 2.70E-04) and cell wall biogenesis (P value = 2.20E-04). The identified molecular functions were ATPase activity (P value = 8.05E-05) and ATP binding (*P* value = 5.09E-05), while the identified cellular component was the cell wall class (*P* value = 9.95E-05). These findings suggest an evolutionary pressure for change in genes associated with cell wall remodeling. The unique lipid-rich mycobacteria cell wall is the first line of interaction between host and pathogen, with crucial implications in virulence, and is also important for environmental stress resistance (e.g. protection from osmotic stress, starvation, freezing, and desiccation).

**Fig. 2:**
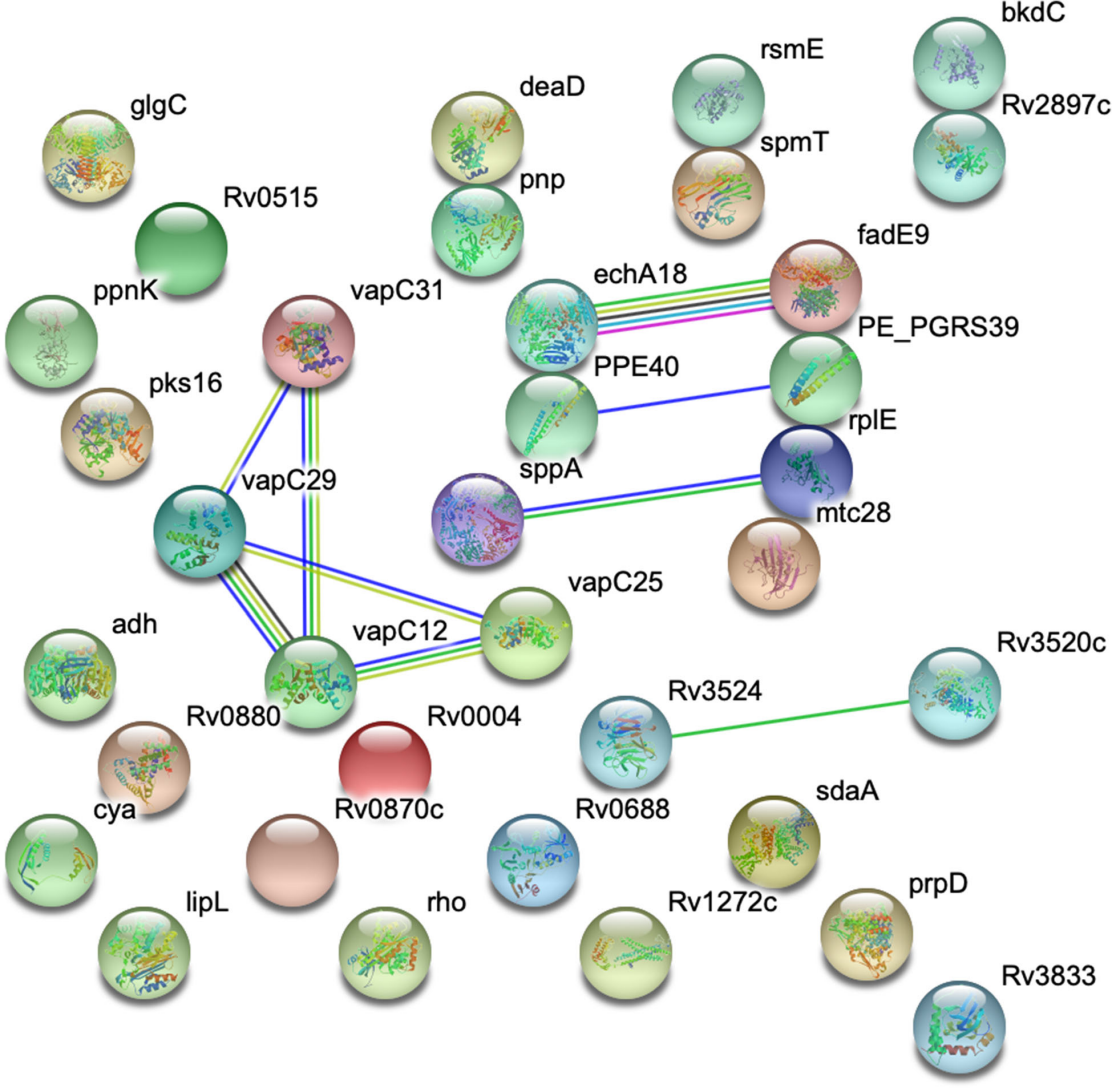
Protein interaction network of CDS (coding DNA sequences) with known function containing non-synonymous SNPs (single nucleotide polymorphisms). CDSs with mutations were identified between strains MP1 and MP2 of *Mycobacterium pinnipedii*. Network was generated using STRING database version 11.0, with default parameters. Green edge: neighborhood evidence; blue edge: co-ocurrence evidence; pink edge: experimental evidence; yellow edge: textmining evidence; black edge: co-expression evidence

When using STRING, we identified strong protein interactions between specific members of the VapBC protein family (Fig. 2). The fact that multiple proteins with STRING-detected relationships have non-synonymous SNPs also indicates that these Vap genes, in particular, are under evolutionary pressure for change. In *M. tuberculosis*, the VapBC family accounts for more than half of the proportion of toxin-antitoxin (TA) systems (47/88 putative TA systems) [21], and genomic regions containing VapBC are strongly associated with virulence and pathogenicity factors [22]. The identified CDSs are part of a type-II TA system related to mRNA translation regulation. More specifically, VapC25 and VapC29 are ribonucleases shown to inhibit *M. smegmatis* cell growth when induced (i.e. have bacteriostatic effects) [23]. Taken together, these results suggest that MP1 and MP2 may have different survival capacities.

Two distinct spoligotypes, SB0155 and SB2455, and two distinct Mycobacterial Interspersed Repetitive Unit-Variable Number of Tandem Repeat (MIRU-VNTR) profiles were identified in *M. pinnipedii* MP1 and *M. pinnipedii* MP2, respectively (Additional file 2: Figures S1 and S2). The in silico predicted MIRU-VNTR matched the pattern identified using PCR. The *M. pinnipedii* strains MP1 and MP2 differed at MIRU04 and MIRU10 loci (Additional file 2: Figure. S1). Variations at multiple loci between isolates of the same animal normally indicate mixed-strain infections [24]. In contrast to the spoligotype SB0155 [25], SB2455 profile has never been described in *M. pinnipedii* strains. Therefore, based on current parameters of distinction between microevolution and superinfection regarding SNPs/indels [26] and MIRU-VNTR [24], the sea lion analyzed herein was infected with two different strains of *M. pinnipedii*.

This is the first report of *M. pinnipedii* superinfection in a sea lion. As these animals normally live in dense colonies, they are highly vulnerable to epizootics of infectious diseases that can be transmitted by direct animal-to-animal contact. Superinfection caused by *M. tuberculosis* in humans is normally reported in highly endemic countries, in which people are exposed to multiple strains of *M. tuberculosis* throughout their lives and HIV plays an important role in shaping the disease incidence [24]. By tracing a parallel, it is possible that *M. pinnipedii* is highly endemic in the population from which this sea lion came, represented by different circulating strains, which may have unprecedented effects on the conservation of this species. Adverse environmental conditions, insufficient nutrition, and chronic stress due to disturbance or competition can act to suppress the immune system and, therefore, pinnipeds may be predisposed to diseases caused by several pathogens. In Brazil, sea lion stranding occurs normally due to compromised animal health when they travel long distances during the migratory period [27]. The weakened state in which these animals arrive at the coast, often infected by large bacterial loads, is the factor that most commonly leads to death [28, 29]. The contribution of *M. pinnipedii* infection in this context remains to be elucidated.

Although *M. pinnipedii* has been described in both captive and free-ranging animals, systematic population surveillance studies are still lacking. In 2011, a captive South American sea lion housed in a Czech Republic zoo that was imported from a German zoo (not specified) was found to be infected with *M. pinnipedii*. The animal’s parents also died of tuberculosis in Germany and they were captured as juveniles from the coastal waters of Uruguay in 1992 [16], suggesting that the infection came from wildlife. Outbreaks of the disease in wild and/or captive-born South American sea lions housed at the Heidelberg Zoo, Germany and the Le Pal Zoo, France have also been described [30]. Added to this report of superinfection, it is thus clear that *M. pinnipedii* is endemic in wild populations of South American sea lions and is being introduced into zoos, warranting an urgent need to evaluate the extent of tuberculosis in free-ranging pinnipeds of the southern hemisphere.

### Strains of *M. pinnipedii* have a highly conserved proteome

**Fig. 3:**
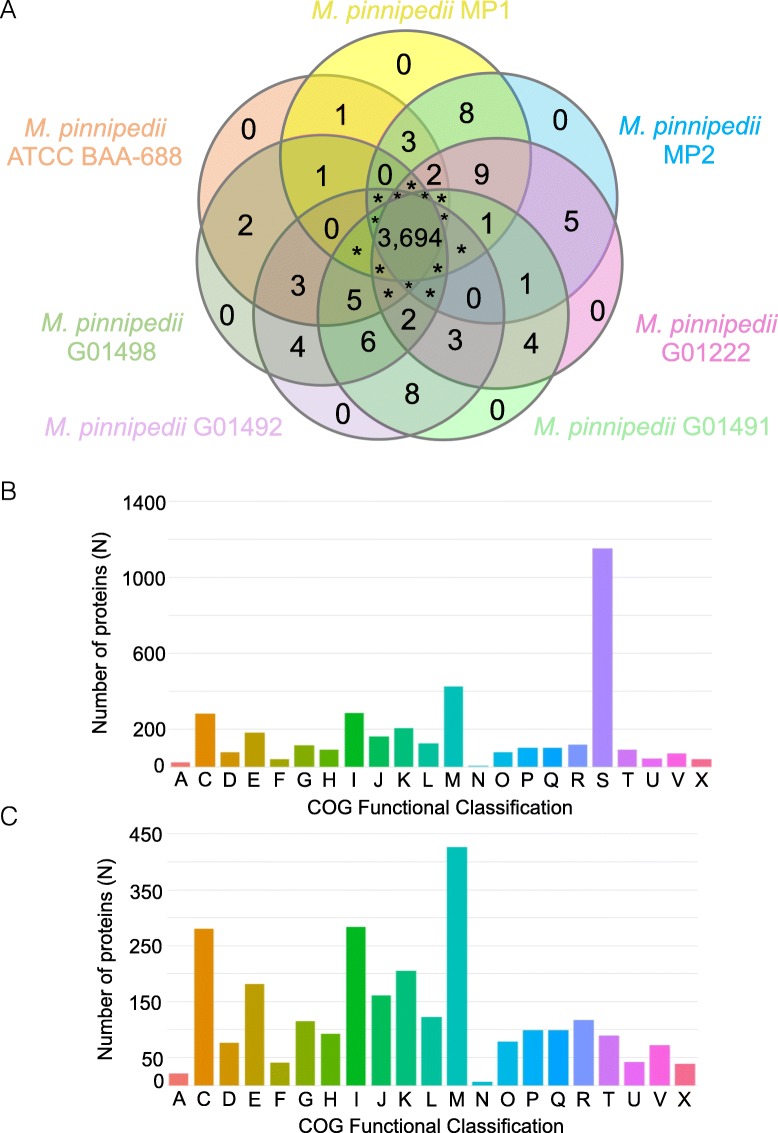
Clusters of orthologous proteins of *Mycobacterium pinnipedii* strains. **a** Venn diagram of orthologous proteins. **b** Cluster of Orthologous Groups (COG) classification based on function of the core protein clusters of the *M. pinnipedii* strains. **c** COG classification without unknown function class (S). Protein clustering was performed using OrthoMCL as available in KBase plataform. *The number of orthologous proteins shared between these groups are available in Additional file 4: Table S3. Cluster size varied from two to forty-three proteins

Figure 3 illustrates groups of orthologous proteins present in seven modern *M. pinnipedii* strains that were deposited in the National Center for Biotechnology Information (NCBI) (*M. pinnipedii* MP1, MP2, ATCC BAA-688, G01222, G01491, G01492, G01498). A total of 3,986 protein clusters (i.e. groups of ≥2 proteins) were identified among the analyzed strains, including 3,694 (92.67%) core protein clusters present in all seven genomes (Fig. 3a; Additional file 3: Table S2). According to COG classification, about a third (1,147/3,694; 31.05%) of the core protein clusters have unknown function, while the majority of these have functional annotation (2,547/3,694; 68.95%). The top five functional classes were: cell wall/membrane/envelope biogenesis (M; 426/3,694, 11.5%), lipid transport and metabolism (I; 284/3,694, 7.7%), energy production and conversion (C; 280/3,694, 7.6%), transcription (K; 205/3,694, 5.5%) and amino acid transport and metabolism (E, 181/3,694, 4.9%) (Fig. 3b-c).

There are no protein clusters (i.e. groups of ≥2 proteins) unique to each *M. pinnipedii* strain (Fig. 3a). However, a number of single proteins not categorized into any protein cluster were considered unique to each *M. pinnipedii* strain (MP1 = 32, MP2 = 37, ATCC BAA-688 = 58, G01498 = 103, G01492 = 68, G01491 = 52, G01222 = 80). As the annotation tool used herein (RAST; see methods) does not differentiate between pseudogenes/broken genes and true genes, we used BLASTp and/or tBLASTn to search the NCBI’s PGAP annotation of MP1, MP2 and ATCC BAA-688 strains using these “unique proteins” as input. From those with annotated function (i.e. not hypothetical or PE/PPE genes), our BLAST search revealed that all functional CDSs are considered pseudogenes, are non-existent or are present in contigs smaller than 700 bp (with doubtful sequencing/assembly quality) based on PGAP annotation (Additional file 5: Table S4). As RAST reports broken CDSs at the end of contigs as true genes, these CDS fragments (i.e. artefacts of draft genomes) failed to cluster into orthologous groups. Nevertheless, there is still a number of hypothetical and PE/PPE genes that we were not able to distinguish between pseudogenes and true genes using the proposed tools, indicating the possibility of distinct phenotypes within *M. pinnipedii* strains. These variations may be a consequence of gene truncation or pseudogenization, which may lead to neofunctionalization or gene loss, respectively, as well as duplication events, phenomena commonly observed in the MTBC [31, 32].

When considering non-core protein clusters (*n* = 292) shared between two and up to six *M. pinnipedii* strains, similar results were obtained, with all functional CDSs identified as pseudogenes or non-existent based on PGAP annotation, and an unknown number of possible hypothetical and PE/PPE genes that may be variably present among *M. pinnipedii* strains. Taken together, our results indicate that the analyzed *M. pinnipedii* strains present a high level of proteome conservation, which is in contrast with a recent pangenome analysis of *M. tuberculosis* strains that detected at least 1,122 CDSs in the accessory genome and 964 strain-specific CDSs in 36 genomes [33]. However, the *M. tuberculosis* pangenome in that study was also analyzed using RAST, and pseudogenes were not taken into consideration.

### Modern *M. pinnipedii* strains have unique deletion markers

**Fig. 4:**
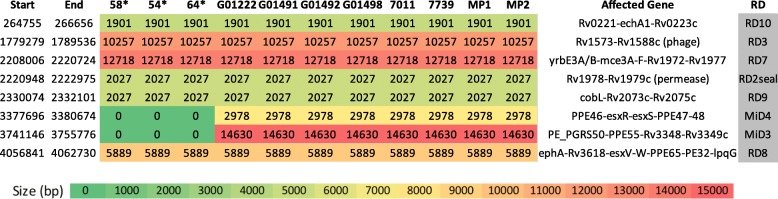
Large sequence polymorphisms (LSPs) of *Mycobacterium pinnipedii* strains. Values indicate number of nucleotides spanning each deleted region (zero means the region is not deleted in the corresponding bacterial strain). Start and end: nucleotide positions according to *Mycobacterium tuberculosis* H37Rv reference genome. Genes are annotated according to reference genome. *Ancient South American *Mycobacterium pinnipedii* strains. RD: regions of difference. All listed deletions have been already described in modern *M. pinnipedii* strains

In the large sequence polymorphism (LSP) analysis, eight previously described deletions (RD2seal, MiD3, MiD4, RD3, RD7, RD8, RD9, RD10) were found in *M. pinnipedii* strains (Fig. 4). Although the genome coverage of the ancient *M. pinnipedii* reads in respect to *M. tuberculosis* H37Rv varied from 31.94 to 47.83% (after quality trimming), the deletions MiD3 and MiD4 were not found in the ancient strains of *M. pinnipedii*, while present in the eight analyzed modern strains of the same species. This finding contributes to the understanding of *M. pinnipedii* evolution over time, indicating an active process of genome reduction occurring for the past one thousand years.

We have also detected 19 regions with ambiguous read mapping (i.e. areas with low sequencing coverage composed of mapped reads with non-specific match) ranging from 548 bp to 5329 bp (Additional file 6: Table S5). The vast majority (16/19; 84.21%) are associated with at least one PE/PPE gene, while the three remaining regions comprise a hypothetical protein with an oxireductase (Rv3530c-Rv3531c) and two transposases (Rv0797 and Rv3023c). Given the repetitive nature of these regions and possible sequencing coverage bias, these findings should be interpreted with caution. Future validation using PCR assays are strongly indicated. The PE/PPE gene families correspond to approximately 10% of the coding capacity of the *M. tuberculosis* genome [34] and show a high degree of variation across members of the MTBC and between strains of the same species.

**Fig. 5:**
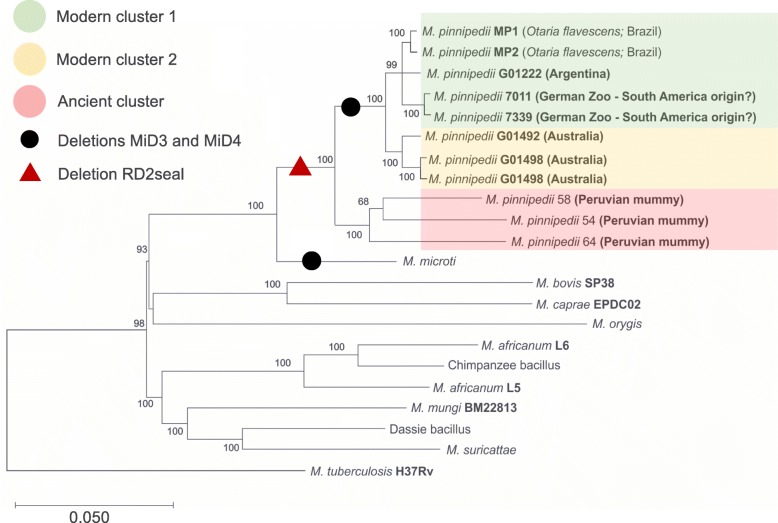
Phylogenetic tree based on SNPs (single nucleotide polymorphisms) of *Mycobacterium tuberculosis* Complex (MTBC) using Maximum Likelihood (ML) model. Green box: modern cluster 1; orange box: modern cluster 2; pink box: ancient cluster; black circle: deletions MiD3 and MiD4; red triangle: deletion RD2seal. *Mycobacterium tuberculosis* H37Rv was used as outgroup. A customized script in Python version 3.6.3 was used to build a matrix of SNPs, which was used to infer a ML tree using RAxML with GTRCAT model and autoMRE for best-scoring ML tree and a maximum of 1000 bootstrap inferences using RaxML software version 7.3

The finding of MiD3 and MiD4 being present only in modern strains of *M. pinnipedii* suggests that these deletions may have occurred independently, twice, over its evolutionary course (Fig. 5). MiD3 and MiD4 were first identified in *Mycobacterium microti* [35, 36]. This pathogen was initially described in voles [37], but has since been isolated from pigs, llamas, cats, wild boars, and immunosuppressed humans, being restricted to Eurasia. This raises questions about the true host range of these closely related pathogens, which may or may not have changed over the past one thousand years.

### Two distinct clusters of modern *M. pinnipedii* strains

Figure 5 illustrates the SNP-based phylogenetic tree of the MTBC including eight modern and three ancient *M. pinnipedii* strains. A total of 1698 polymorphic positions were found to be unique to *M. pinnipedii* strains, i.e. not present in any other of the MTBC species included in this study (Additional file 7:Table S6). This finding is not comprehensive of all possible MTBC strains distributed worldwide, which means that different strains not analyzed herein may also have mutations in these same polymorphic sites. In addition, strains of *M. pinnipedii* appeared distributed among three main clusters, with the ancient strains emerging from the most basal node (Fig. 5). Interestingly, modern *M. pinnipedii* strains appeared divided into two groups according to geographic locations, and possibly host species: modern cluster 1, comprising isolates of South American origin, and modern cluster 2, comprising isolates from Australia. These results are in accordance with findings of *M. tuberculosis* and *M. bovis* lineages/clonal complexes that are also associated with distinct geographic locations [32, 38, 39]. Whether or not these are also associated with different virulence phenotypes is unknown.

## Conclusion

This is the first report of mixed-strain infection of *M. pinnipedii* in pinnipeds. This finding, coupled with previous reports in the literature, suggest that *M. pinnipedii* infection is endemic in free-ranging populations of South American sea lions. Genetic differences between these strains were found to be associated with virulence factors and enzymes necessary for intracellular maintenance and membrane-shaping of mycobacteria. The actual effect of these phenomena for the disease outcome and conservation of the animal species, and potential population-level implications are unknown. Nevertheless, this prompts an urgent need to evaluate the extent of the disease in these animals and to consider tuberculosis in pinnipeds as one of the main health concerns for these species. Investigations of the infection and clinical disease should be conducted when introducing animals into zoo facilities and studies involving free-ranging populations are encouraged.

As with other species of MTBC, *M. pinnipedii* genomes are under evolutionary decay through the loss of specific genomic regions. These results were further supported by the finding of LSPs not present in the ancient genomes of *M. pinnipedii* compared to modern *M. pinnipedii* strains. The genome remodeling of *M. pinnipedii* affects well-known MTBC virulence factors, with potential impact on host adaptability and disease outcome, such as the deletion of MiD3 and MiD4 and possible regions containing PE/PPE genes. However, the proteome of the studied strains showed high degree of conservation, indicating that further genomes need to be sequenced and analyzed to increase the chance of finding significant differences or that *M. pinnipedii* genome remodeling occurred prior to bacterial speciation. In addition, particular attention should be given to possible “pseudogenes” and/or truncated CDSs, as to standardize genome annotations and guide future gene-based analyses. And finally, *M. pinnipedii* strains, as with other MTBC species, are likely to cluster based on geographic occurrence, which will coincide with pinniped species, as these animal populations are already segregated by geography.

## Methods

### Selection of genomes

Sequence reads from six modern *M. pinnipedii* strains [G01222 from Argentina; G01491, G01492, G01498 from Australia; and 7739 and 7011 from a zoo in Germany (SRR1239336, SRR1239337, SRR1239338, SRR1239339, SRR1239341, and SRR1239340)] and three ancient *M. pinnipedii* strains from 1000-year-old Peruvian mummies were selected for this study [58, 54 and 64 (SRR1238557, SRR1238558, and SRR1238559)] [5]. All read sets were retrieved from the Sequence Read Archive (SRA) of the NCBI. Assembled contigs of *M. pinnipedii* strain ATCC BAA-688 (NZ_MWXB01000000) from Australia deposited in RefSeq were also selected [20]. Read sets from this ATCC strain (BAA-688) are not deposited in public databases and were not included in any analysis requiring reads. Also, *Mycobacterium pinnipedii* strains 7739 and 7011 had only single read sets publicly available, and these were not appropriate for genome assembly and not included in analyses requiring assemblies (Table 1). Whole genome of *M. tuberculosis* H37Rv (NC_000962.3) was used as reference. For the phylogenomic analysis, reads of MTBC representatives [chimpanzee bacillus (ERR150046), dassie bacillus (SRR3745458), *M. africanum* GM041182 (ERR234255), *M. africanum* MAL010070 (SRR998578), *M. bovis* SP38 (SRR6705904), *M. caprae* EPDC02 (DRR120409), *M. microti* 94–2272 (ERR027298), *M. mungi* BM22813 (SRR3500411), *M. orygis* IDR1100020842 (SRR5642712), *M. suricattae* ERS798580 (ERR970409)] were also included.

**Table 1:** Assembly statistics, genotyping and demographics of modern *Mycobacterium pinnipedii* strains used in this study

Strains	Assembly statistics						Genotyping	Demographics	
	Available data	Coverage	Number of contigs	N50	Contig size (range)	Software	Spoligotypes	Source (species, country)^a^	Ref.
*M. pinnipedii* MP1	Paired-end reads and draft genome	228x	102	61,148	1020-184,975	CLC Genomics Workbench 11	SB0155	*Otaria flavescens*, Brazil	This study
*M. pinnipedii* MP2	Paired-end reads and draft genome	220x	106	60,933	1010- 184,294	CLC Genomics Workbench 11	SB2455	*Otaria flavescens*, Brazil	This study
*M. pinnipedii* G01222	Paired-end reads	32x	138	38,346	1020-121,089	CLC Genomics Workbench 11	SB0155	Argentina	[5]
*M. pinnipedii* G01491	Paired-end reads	242x	194	45,251	1135–150,198	SPAdes 3.13.0	SB0155	Australia	[5]
*M. pinnipedii* G01492	Paired-end reads	484x	128	64,538	1146–193,250	SPAdes 3.13.0	SB0155	Australia	[5]
*M. pinnipedii* G01498	Paired-end reads	113x	201	42,094	1026-122,203	SPAdes 3.13.0	SB0155	Australia	[5]
*M. pinnipedii* 7739^b^	Single reads	86x	ND	ND	ND	ND	SB0155	Germany (zoo)	[5]
*M. pinnipedii* 7011^b^	Single reads	87x	ND	ND	ND	ND	SB0155	Germany (zoo)	[5]
*M. pinnipedii* ATCC BAA-688	Draft genome	NA	162	113,505	500–281,603	Velvet 1.2.10	Unknown^c^	Australia	[20]

ND: not done. NA: not available. ^a^where animal species are not listed, it is because they were not informed by the authors. ^b^Reads of *M. pinnipedii* 7739 and 7011 were not assembled because they were available as single reads. These reads were only used in the phylogenomic analysis. ^c^Spoligotype pattern not found in Mbovis.org database (Bincode: 0000001000000000000000010100010001000000000). Sequencing coverage was calculated by the number of bases (after adaptors removal and quality trimming) divided by the average size of an MTBC genome (i.e. 4.3 Mb). Spoligotypes were defined using SpoTyping [40]

### Isolation and bacterial DNA extraction

Two *M. pinnipedii* isolates (MP1 and MP2) obtained from the lung and a mesenteric lymph node of a South American sea lion (*Otaria flavescens*) found dead in Capão da Canoa, Rio Grande do Sul, Brazil [19] were kept at − 80 °C in 7H9 broth with 20% glycerol. For this study, both isolates were reactivated in Stonebrink media and a single colony from each isolate was sub-cultured for DNA extraction as described previously [41, 42]. The quality and quantity of the DNA was measured by Nanodrop 2000c (Thermo Fisher Scientific, Waltham, MA, USA) following the absorbance ratios of 260/280 and 260/230 and in 0.8% agarose gel with a mass ladder. A final analysis was performed with an Agilent 2100 Bioanalyzer High Sensitivity Chip DNA (Agilent Technologies, Santa. Clara, CA, USA) for sample concentration and fragmentation. All procedures involving infectious material were performed in a Biosafety Level 3+ Laboratory (BSL-3+ Prof. Dr. Klaus Eberhard Stewien) located at the Department of Microbiology, Institute of Biomedical Sciences, University of São Paulo, Brazil.

### Sequencing of *M. pinnipedii* isolates

A paired-end genomic library was constructed using a TruSeq DNA PCR-free sample preparation kit (Illumina, San Diego, CA, USA) according to the manufacturer’s instructions. HiSeq2500 with Illumina v3 chemistry was used to sequence the genomic library (100 bp). These procedures were performed at the Central Laboratory of High Performance Technologies in Life Sciences (LaCTAD), State University of Campinas (UNICAMP), Campinas, Brazil. Illumina sequencing reads were deposited in the SRA, NCBI under accession numbers: SRR7693584 and SRR7693090.

### Assembly and annotation of the genomes

The 100 bp paired-end reads from each library were first filtered by quality and presence of adaptors using the Trimmomatic software version 0.36 [43]. *Mycobacterium pinnipedii* MP1 and MP2 genomes were de novo assembled using the SPAdes software version 3.13.0 [44]. Gene identification and annotation were performed automatically by the NCBI’s PGAP and draft genomes deposited as GCA_003027795.2 (MP1) and GCA_003027895.2 (MP2).

### Circular map of *M. pinnipedii* genomes

In order to visualize the similarity among the predicted proteins of different *M. pinnipedii* strains, the genomes of strains G01222, G01491, G01492 and G01498 were assembled using CLC Genomics WorkBench 11 (Qiagen, Netherlands) and SPAdes software version 3.13.0 [44]. The best assemblies based on the number of contigs and N50 were selected (Table 1). Assembled contigs were then annotated with RAST version 2.0 [45]. Resulting contigs from strains G01222, G01491, G01492 and G01498 and contigs from *M. pinnipedii* strains MP1, MP2, and ATCC BAA-688 deposited in GenBank were reordered with MAUVE [46] using *M. tuberculosis* H37Rv (GenBank NC000962.3) as the reference genome. Subsequently, a circular genomic map was constructed using the Proteome Comparison toll implemented in Patric 3.5.18 web resources [47].

### Mapping of reads and variant calling

The Burrows-Wheeler Aligner (BWA) software version 0.7.17 – r1188 [48] was used to map the reads of *M. pinnipedii* MP1 and MP2 against the reference genome *M. tuberculosis* H37Rv, separately, and the reads of *M. pinnip*e*dii* MP2 against the assembled contigs of *M. pinnipedii* MP1. The BWA outputs were analyzed with SAMtools version 1.9 [49] to sort and index .bam files, according to filters of mapping quality of Phred’s scale 20 and removal of duplicated reads. Detection of SNPs and insertions and deletions (indels) were performed with FreeBayes version 1.3.1–1 [50] with the following parameters: minimum mapping quality of 10, minimum base quality at a position of 10, minimum read depth at a position of 5 and without strand bias. The maximum indel (insertion or deletion) size reported by FreeBayes was 36 bp. Identified SNPs and indels were then annotated using SNPEff version 4.0 [51]. Variants annotated in regions related to transposable elements, 13E12 family, phages or PE/PPE family of proteins were removed to avoid false positives.

To identify unique SNPs of *M. pinnipedii*, reads of MTBC representatives (chimpanzee bacillus, dassie bacillus, *M. africanum* GM041182, *M. africanum* MAL010070, *M. bovis*, *M. caprae*, *M. microti*, *M. mungi*, *M. orygis*, *M. suricattae*) were included in the analysis, mapped against *M. tuberculosis* H37Rv and variants were called. The variants present only in the 11 strains of *M. pinnipedii*, and not in any other of the analyzed MTBC species, were identified.

### Protein interaction networks and GO enrichment analysis

CDSs identified with non-synonymous mutations comparing MP1 and MP2 strains were analyzed using STRING database version 11.0 with default settings for the prediction of network associations between proteins [52]. Results were used to identify specific metabolic pathways or protein interactions under evolutionary pressure. We also performed a GO (Gene Ontology) enrichment analysis (biological processes, molecular function and cellular component) of the same CDSs using PANTHER version 14 according to *M. tuberculosis* H37Rv reference annotation [53]. Results were considered significant when *P* value ≤0.05. When needed, protein sequences were searched against the Virulence Factor Database (VFDB) [54] to identify virulence factors.

### In silico spoligotyping and mycobacterial interspersed repetitive unit-variable number of tandem repeat (MIRU-VNTR)

For the identification of spoligotypes, the reads of *M. pinnipedii* isolates were analyzed in SpoTyping version 2.0 [40]. The identified patterns were submitted to the Spoligotyping database of *M. bovis* (www.mbovis.org) for the identification of the pattern number. In order to identify the variable number tandem repeats (VNTRs) of genetic elements named mycobacterial interspersed repetitive units (MIRUs), the 24 MIRU-VNTR loci were individually identified in silico using previously described primers [55] and the resulting patterns were analyzed in the MIRU-VNTRplus database [56, 57]. We also performed 24-loci MIRU-VNTR PCR using extracted DNA from *M. pinnipedii* MP1 and *M. pinnipedii* MP2 as previously described [55]. PCR products were separated by electrophoresis in a 1.5% agarose gel stained with SYBR Safe DNA Gel Stain (Thermo Fisher Scientific) and visualized under ultraviolet light.

### *Mycobaterium pinnipedii* orthologous and paralogous protein clusters

To identify groups of orthologous genes, seven *M. pinnipedii* strains were used (G01222, G01491, G01492, G01498, MP1, MP2 and ATCC BAA-688). The *M. pinnipedii* strains G01498, G01492, G01491 and G01222 are not available as assembled genomes in GenBank, which precluded PGAP annotation. As to avoid biases in orthology clustering due to discrepancies in annotation from different platforms, we then annotated or re-annotated all genomes analyzed herein using RAST version 2.0 [45]. Predicted CDS ≤150 bp were excluded. Proteins were then clustered using OrthoMCL [58] available from the KBase platform [59]. Briefly, homologous pairs of sequences were found using the all-against-all BLASTp algorithm with an E-value <1e-5. OrthoMCL then converted the BLASTp results into a normalized similarity matrix that was analyzed by a Markov Cluster algorithm (MCL) for clustering of orthologous sequences. The inflation index of 1.5 was used to regulate cluster tightness. Since the RAST algorithm reports pseudogenes and broken genes located at the end of contigs as true genes, the number of strain-specific CDSs may be overestimated. This overestimation was manually checked using BLASTp and/or tBLASTn [60] by comparing obtained results with the annotation of *M. pinnipedii* strains MP1, MP2 and ATCC BAA-688, which are deposited in Genbank and have been annotated with PGAP from NCBI. Groups of orthologous proteins were classified with eggNOG [61] using a graph-based unsupervised clustering algorithm extending the COG methodology [62].

### Large sequence polymorphisms

LSPs were detected with CLC Genomics Workbench 11 (QIAGEN, Venlo, Netherlands) by mapping reads of ancient *M. pinnipedii* (samples 54, 58, 64) and modern *M. pinnipedii* (G01222, G01491, G01492, G01498, 7739, 7011, MP1, MP2) strains against *M. tuberculosis* H37Rv. When mapping the ancient samples against the reference genome, special treatment was given to the reads prior to mapping. Specifically, reads were trimmed based on quality parameters of 0.05 base-calling error probability and presence of two or more ambiguous nucleotides at the ends using modified-Mott trimming algorithm. Reads were mapped against the reference genome in two ways: by excluding or not non-specific matches. A non-specific match is given when a “read aligns at more than one position of the genome with an equally good score”. Obtained results were subsequently compared and contrasted. In both scenarios, mapped regions with less than 10 aligned reads and/or containing low mapping quality reads were considered absent in the query genome. LSPs were called when this region spanned more than 500 bp.

### Phylogenetic analysis

Members of MTBC and eleven of the *M. pinnipedii* strains described above were used to construct a phylogenetic tree. Briefly, all read sets were mapped against the reference *M. tuberculosis* H37Rv genome and true variants were identified as described above, with exclusion of SNPs located in repetitive regions (transposable elements, 13E12 family, phages or PE/PPE family of proteins). A customized script in Python language (software version 3.6.3) was then used to build a positional matrix of SNPs identified in genomes of all strains/species. The matrix was used as input for the RAxML software version 7.3 [63] for the construction of the phylogenetic tree using Maximum Likelihood (ML) algorithm with GTRCAT model and autoMRE for best-scoring ML tree and a maximum of 1000 bootstrap inferences.

## Supplementary information

**Additional file 1: Table S1.** Single nucleotide polymorphisms (SNPs) and indels that differentiate *Mycobacterium pinnipedii* strains MP1 and MP2.

**Additional file 2: Figure S1.** MIRU-VNTR and spoligotype of *Mycobacterium pinnipedii* strains MP1 and MP2. **Figure S2.** PCR of loci MIRU04 and MIRU10 of *Mycobacterium pinnipedii* strains MP1 and MP2.

**Additional file 3: Table S2.** Clusters of orthologous proteins between strains of *Mycobacterium pinnipedii*.

**Additional file 4: Table S3.** Number of clusters of orthologous proteins between strains of *Mycobacterium pinnipedii*.

**Additional file 5 Table S4.** Clusters of orthologous proteins specific of *Mycobacterium pinnipedii* strains MP1, MP2 and ATCC BAA-688.

**Additional file 6: Table S5.** Large sequence polimorphisms (LSP) among *Mycobacterium pinnipedii* strains - candidates that need further experimental validation.

**Additional file 7: Table S6.** Unique variants found in strains of *Mycobacterium pinnipedii* compared to other strains of the *Mycobacterium tuberculosis* complex.

## Data Availability

The datasets generated during the current study are available in RefSeq and SRA: https://www.ncbi.nlm.nih.gov/assembly/GCA_003027895.2, https://www.ncbi.nlm.nih.gov/assembly/GCA_003027795.2, https://www.ncbi.nlm.nih.gov/sra/?term=SRR7693584, https://www.ncbi.nlm.nih.gov/sra/?term=SRR7693090.

## Abbreviations

CDSs: Coding DNA Sequences
COGs: Clusters of Orthologous Groups
Indels: Insertions and/or deletions
LSP: Large Sequence Polymorphisms
MIRU-VNTR: Mycobacterial Interspersed Repetitive Unit-Variable Number of Tandem Repeat
MTBC: *Mycobacterium tuberculosis* Complex
NCBI: National Center for Biotechnology Information
PE/PPE: Proline-Glutamate and Proline-Proline-Glutamate
PGAP: Prokaryotic Genome Annotation Pipeline
RDs: Regions of difference
SNPs: Single nucleotide polymorphisms
SRA: Sequence Read Archive
TA: Toxin-antitoxin
