# Supplementary material for: Genome sequencing of Mycobacterium pinnipedii strains: genetic characterization and evidence of superinfection in a South American sea lion (Otaria flavescens)
Source: BMC Genomics. 2019 Dec 30;20:1030. doi: 10.1186/s12864-019-6407-5 (PMC6937676; doi:10.1186/s12864-019-6407-5)
Supplement: Supplementary file 2 — Additional file 2: Figure S1. MIRU-VNTR and spoligotype of Mycobacterium pinnipedii strains MP1 and MP2. Figure S2. PCR of loci MIRU04 and MIRU10 of Mycobacterium pinnipedii strains MP1 and MP2. [file 12864_2019_6407_MOESM2_ESM.pdf]

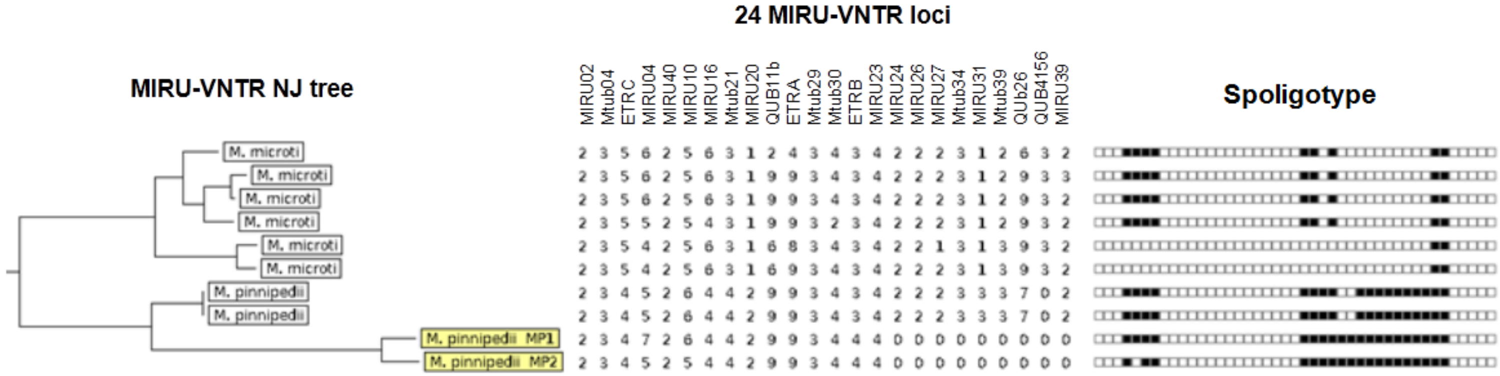


**Figure S1. MIRU-VNTR and spoligotype of *Mycobacterium pinnipedii* strains MP1 and MP2.** A MIRU-VNTR-based dendrogram was generated using Neighbor joining (NJ) tree analysis. *Mycobacterium pinnipedii* strains MP1 and MP2 (yellow boxes) were identified using the MIRU-VNTRplus (http://www.miru-vntrplus.org) identification database. 24 loci MIRU-VNTR alleles and spoligotypes of *Mycobacterium microti* isolates are also represented. MIRU-VNTR: mycobacterial interspaced repetitive units – variable number of tandem repeats.

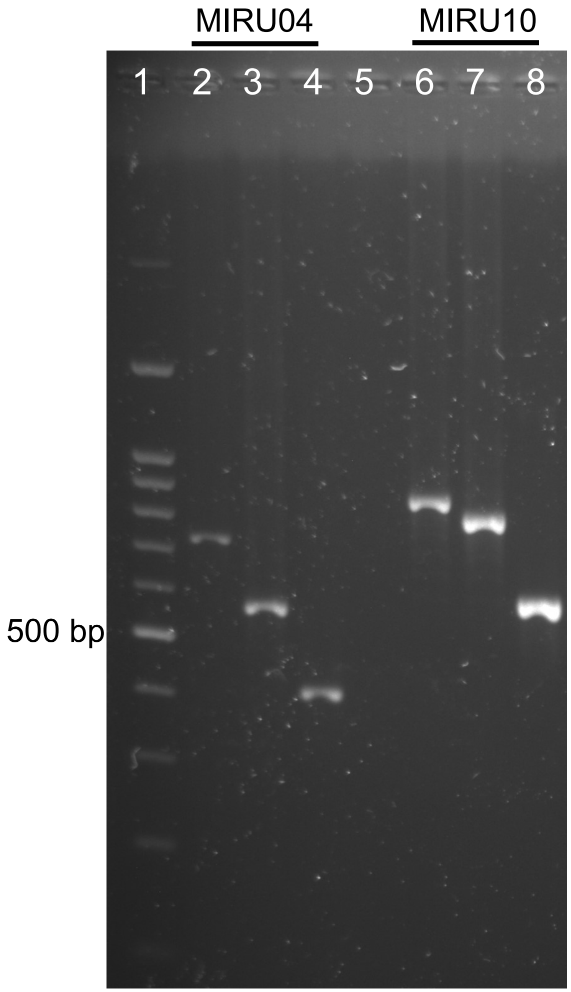


**Figure S2. PCR of loci MIRU04 and MIRU10 of *Mycobacterium pinnipedii* strains MP1 and MP2.** SYBR safe-stained agarose gel of MIRU-VNTR PCR products. Lane 1: Molecular weight marker (100bp, DNA ladder, ThermoFisher Scientific). Lanes 2 to 4: MIRU04 locus. Lane 3: negative control (ultrapure water). Lanes 6 to 8: MIRU10 locus. Lanes 2 and 6: *Mycobacterium pinnipedii* MP1. Lanes 3 and 7: *M. pinnipedii* MP2. Lanes 4 and 8: positive control (*Mycobacterium bovis* AN5). According to Supply et al., 2005, MIRU04

alleles of *M. pinnipedii* MP1 and MP2 are 7 (714 bp) and 5 (560 bp), respectively, and MIRU10 alleles of *M. pinnipedii* MP1 and *M. pinnipedii* MP2 are 6 (802 bp) and 5 (749 bp). MIRU-VNTR: mycobacterial interspaced repetitive units – variable number of tandem repeats. All 24 MIRU-VNTR loci were run; only the loci showing differences (MIRU04 and MIRU10) between the *M. pinnipedii* strains are shown for convenience.
